# Enhanced specific loss power of hematite–chitosan nanohybrid synthesized by hydrothermal method

**DOI:** 10.1098/rsos.230384

**Published:** 2023-10-11

**Authors:** Nandita Deb, Rimi Rashid, H. Das, Ishtiaque M. Syed, S. Manjura Hoque

**Affiliations:** ^1^ Materials Science Division, Atomic Energy Centre Dhaka, Dhaka 1000, Bangladesh; ^2^ Department of Physics, Dhaka University, Dhaka 1000, Bangladesh

**Keywords:** hematite, hyperthermia, morin transition, magnetization, HRTEM

## Abstract

We used a hydrothermal technique to develop nano-scale α-Fe_2_O_3_ particles and functionalized them with chitosan. An X-ray diffraction study revealed α-Fe_2_O_3_ nanoparticles were of single-phase, lattice constants were *a* = 5.07 Å and *c* = 13.68 Å, and the grain size was 27 nm. The presence of lattice fringes in the HRTEM image confirmed the crystalline nature of the α-Fe_2_O_3_. The Mössbauer spectra reveal a mixed relaxation state, which supports the PPMS studies. Zero-field cooled studies revealed the existence of a Morin transition and blocking temperature. The z-average value of the coated particles by DLS was between 218 and 235 nm, PDI ranged from 0.048 to 0.119, and zeta potential was +46.8 mV. We incubated the Vero and HeLa cell lines for 24 h to study the viability of the nanohybrids at different concentrations. Hyperthermia studies revealed the maximum temperature and specific loss power attained by the hematite–chitosan nanohybrid solution of a concentration between 0.25–4 mg ml^−1^. The *T*_max_ at the lowest and highest concentrations of 0.25 and 4 mg ml^−1^ were 42.9 and 48.3°C, while the SLP were 501.6 and 35.5 W g^−1^, which are remarkably high when the maximum magnetization of α-Fe_2_O_3_ nanoparticles was as small as 1.98 emu g^−1^ at 300 K.

## 1. Introduction

Cancer is a group of diseases involving a cluster of cells undergoing abnormal growth in the body [1]. One of the promising approaches to destroying tumours is magnetic hyperthermia. Hyperthermia is a type of cancer treatment in which malignant tumour tissue is exposed to temperatures, typically 42–46°C. Gilchrist *et al*. first suggested the treatment of cancer by hyperthermia using magnetic nanoparticles [1], which are still under development today [2–5].

In the literature, there are numerous reports on the different forms of iron oxides, such as *α*- Fe_2_O_3_, *β*-Fe_2_O_3_, *γ*-Fe_2_O_3_ and *ε*-Fe_2_O_3_ because of their fascinating fundamental properties and plausible technological applications [6–9]. Among all these, α-Fe_2_O_3_ (hematite) nanoparticles have attracted considerable attention in biomedical applications due to their abundance, low cost, low toxicity, excellent chemical stability and tunable optical and magnetic properties. Hematite has a corundum crystal structure. The iron atom has a magnetic moment due to four unpaired electrons in its 3d orbitals. Hematite nanocrystals might possess different magnetic states depending on their size and morphology. In the paramagnetic state the individual atomic magnetic moments align randomly, and the crystal has a zero net magnetic moment. Under an external magnetic field, some of these moments will align, and the crystal will attain a small net magnetic moment.

Hematite is weakly ferromagnetic at room temperature. The Néel temperature for bulk hematite is at *T*_N_ ≈ 960 K. In bulk hematite form, the Morin transition appears at T*_M_* ≈ 263 K [10]. The material shows weak ferromagnetism above *T*_M_ and is antiferromagnetic below *T*_M_. Previous literature reports that by reducing the particle size, the Morin temperature decreases and tends to vanish for particles lower than 10 nm. The hematite nanoparticles exhibited superparamagnetic behaviour above the blocking temperature, *T*_B_, and ferromagnetic below *T*_B_ [10]. As hematite nanoparticles retain antiferromagnetic, weak-ferromagnetic, and superparamagnetic properties, it is a fascinating material for fundamental study. Again, the superparamagnetic properties of hematite might enable us to tailor their anisotropy and magnetization for possible hyperthermia applications. Iron oxide, especially magnetite, attracted much attention for biomedical applications because of its biocompatibility. Reference Daily Intake of Fe is 15 mg compared to Cu approximately 2 mg and Ni < 1 mg. Hematite received much less attention than magnetite as an effective mediator for hyperthermia applications.

The magnetic behaviour of hematite depends on crystallinity, particle size, and the cation substitution [9,11–13]. Below the Morin Transition *T*_M_, the bulk hematite, α-Fe_2_O_3_, becomes antiferromagnetic because two sublattices are oriented exactly along the rhombohedral axis. Magnetization above Morin Transition *T*_M_ demonstrates weakly ferromagnetic properties because of canted sublattice magnetization and uncompensated spins. With the decrease of particle size smaller than 20 nm, *T*_M_ becomes smaller than 4 K, while the effect of the superparamagnetic blocking temperature, *T*_B_, becomes prominent. The particles are ferromagnetic below *T*_B_ and undergo superparamagnetic relaxation above. Magnetic anisotropy can be expressed as,

1.1 $$E(\theta )=KV\sin^{2}\theta .$$

In the above equation, *E* is magnetic anisotropy energy, *K* is the anisotropy constant, *V* is the particle volume, and *q* is the angle between the easy direction of magnetization and the magnetization vector. The energy undergoes two minima at 0 and 90°, which implies that at the energy barrier, *KV* becomes comparable to thermal energy, and superparamagnetic relaxation occurs between these two energy minima [14].

The crystal structure of hematite is of low symmetry, which leads to the magnetocrystalline anisotropy difference along and out of the basal plane. At the nanoscale, surface, and shape anisotropy become significant. They also demonstrated that the particle size from 25 to 6 nm decreases the anisotropy constant ten times. The combined effect of anisotropy and superparamagnetic relaxation emanating from a wide range of particle sizes might be interesting to explore their contribution to the specific loss power of hyperthermia [15]. Therefore, the use of hematite (*α*-Fe_2_O_3_) is investigated as the media for hyperthermia treatment because it might allow better control of magnetic properties than magnetite for hyperthermia applications [15–17].

Magnetic nanoparticles for biomedical applications cannot be applied directly without surface modification [18]. With a proper surface coating, magnetic nanoparticles can be dispersed in a suitable solvent to form a homogeneous suspension for applications in physiological conditions. Chitosan-coating on the hematite surface enabled to achieve biocompatibility. Chitosan is a biocompatible and biodegradable polymer. Its d-glucosamine and B-acetyl-d-glucosamine linked by b-(1.4-glycosidic bonds) provide one free amino group and two free hydroxyl groups in the polymeric chain [19]. Thus, chitosan-coated nanoparticles have a high potential for further drug loading and surface modification. The cationic charge of chitosan can transport a drug to an acidic environment, where the chitosan will degrade, releasing the drug to the targeted tissue. Further, the charged surface due to chitosan coating develops electrostatic repulsion and forms a stable suspension in the water.

In the last decades, the focus of the research has been on synthesizing iron oxide, and many reports have described efficient synthesis approaches to produce shape-controlled, stable, biocompatible and monodispersed iron oxide nanoparticles. The most common methods are chemical co-precipitation [20] sol–gel synthesis [21,22], microemulsion [23–25], thermal decomposition [26,27] and hydrothermal method [28,29]. Hydrothermal synthesis is considered one of the most promising and effective techniques. In the hydrothermal synthesis, maneuvering the pressure, temperature, reaction time, solution pH value, reactant concentration and solvent determine the morphology and crystallinity.

Magnetic properties of nanoparticles are strongly related to their size, pH and crystallinity of materials [30,31]. Since hyperthermia is a promising therapeutic modality for cancer treatment [32,33], it is customary to tune the size of the nanoparticles by varying their physico-chemical parameters. Early works demonstrate that hydrothermal synthesis of maghemite particles enables tuning of the physico-chemical properties by adjusting the pH [34]. The heating efficiency of the nanoparticles was satisfactory under an alternating magnetic field. The increase of frequency from 110 to 523 kHz increases the temperature of the colloids significantly [35,36].

Cancer therapy modalities such as chemotherapy and radiotherapy have potential side effects [37,38] because these therapies affect not only the cancer cells but healthy cells. Targeted and localized therapy will be more efficient and less toxic for cancer treatment by targeting cancer tissue only. Magnetic nanoparticle-mediated hyperthermia can target cancer tissues only, in which magnetic particles transported to a tumour site generate heat that will selectively kill tumour tissue. Cancer cells by magnetic fluid hyperthermia can target the cancer cells selectively because of the unorganized and leaky vasculature of the cancer tissues [39]. Further, tumour tissues do not have functional lymphatic drainage. Therefore, nanoparticles transported to the tumour tissue are trapped inside the tumour by enhanced permeability and retention effect (EPR) [39]. Hyperthermia can be realized as a stand-alone therapeutic protocol or with chemo/radiotherapy. Nanoparticles can be functionalized and prepared to load chemo/radiotherapy drugs on the surface and released to tumour sites with the heat generated by hyperthermia. The heat generated by hyperthermia might weaken cancer cells, which reduces the requirement of the dose needed for chemo/radiotherapy. Our motivation was to continuously search for an excellent mediator that would be of low cost and bear optimum properties for the potential applications in hyperthermia with fewer side effects compared to existing cancer treatments—hematite is the most stable iron oxide compared to magnetite with a high resistance to corrosion, low cost, and it is also biocompatible, environmentally friendly and non-toxic [37,38].

In this research, α-Fe_2_O_3_ nanoparticles were synthesized via the hydrothermal method and coated with chitosan. We found novel hydrothermal synthesis resulted in pristine *α*-Fe_2_O_3_ (hematite) nanoparticles with weak properties yet very intriguingly resulted in a high hyperthermia temperature and specific loss power suitable for α-Fe_2_O_3_ (hematite) mediated drug delivery and hyperthermia.

## 2. Experimental procedures

### 2.1. Material and method

We bought Ferric nitrate (Fe(NO_3_)_3_.9H_2_O, 98.0%) and ferrous chloride (FeCl_2*.*_10H_2_O, 98.0%) from Loba Chemie, India, ammonia (NH_4_OH, 99.9%) from Merck Specialties Pvt Ltd, India, and sodium dodecyl sulfate (NaC_12_H_25_SO4) from Qualikems Fine Chem Pvt Ltd, India. Additionally, we purchased chitosan (75–85% deacetylated) from Sigma-Aldrich, Germany, and Acetic Acid (CH_3_COOH) from Unichem Inc., USA.

In this study, we synthesized iron oxide (α-Fe_2_O_3_) nanoparticles by the hydrothermal method illustrated by Kambiz Hedayati *et al*. with some modifications [40]. The two primary segments of a hydrothermal reactor are an inner Teflon liner or Teflon chamber and an outside high-quality stainless steel jacket. At first, 0.01 M of FeCl_2_.10H_2_O, 0.02 M of Fe(NO_3_)_3_.9H_2_O, and 0.7 g of surfactant (sodium dodecyl sulfate) dissolved in 100 ml of distilled water, followed by the slow addition of 10 ml of NH_3_ solution of concentration 1 M. All the reactants were transferred into the Teflon-lined autoclave of 500 ml capacity and placed into an oven. Hematite nanoparticles evolved at 200°C for 4 h, where the following reaction took place:

$$\begin{array}{rl} {\mathrm{FeCl}}_{2}.10H_{2}O+ & 2\mathrm{Fe}{\left({\mathrm{NO}}_{3}\right)}_{3}.9H_{2}O+8{\mathrm{NH}}_{4}\mathrm{OH}\to {\mathrm{Fe}}_{2}O_{3}\\ +\mathrm{Fe}{\left(\mathrm{OH}\right)}_{2}+2{\mathrm{NH}}_{4}\mathrm{Cl}+6{\mathrm{NH}}_{4}{\mathrm{NO}}_{3}+32H_{2}O. \end{array}$$

Next, the product was centrifuged at 12 000 rpm, washed ten times with distilled water, and dried in the ambiance. Finally, as-synthesized particles were grounded in an agate mortar and pestle for 6 hours.

### 2.2. Coating procedure

We produced a 2% w/v chitosan solution by mixing 0.8 g solid chitosan with 40 ml of distilled water. The mixture was then agitated at a rate of 500 rpm. Chitosan is not water-soluble as such. Therefore, we added approximately 4 ml of acetic acid to the mixture and stirred for 48 h until the chitosan became thoroughly soluble. Further, we centrifuged the solution at 12 000 rpm for 10 min to remove any remnants of solid chitosan. We decanted the homogeneous layer and discarded the bottom layer. We repeated the centrifugation and decantation thrice and stored them as the stock solution.

To produce a chitosan–hematite nanohybrid, 240 mg of the as-dried sample was transferred to a falcon tube. We added 12 ml of chitosan solution dropwise to the falcon tube to yield a chitosan–hematite nanohybrid solution of a concentration of 20 mg ml^−1^. We noticed a change in the appearance of hematite nanoparticles from particulate to colloid with the subsequent addition of the chitosan dropwise. To achieve a complete suspension of hematite particles, we stirred for 20 min at 500 rpm, followed by 20 min of ultrasonication. We then vortexed the solution for 20 min and repeated vortexing and sonication until the completion of functionalization. We achieved appropriate concentrations, e.g. 4, 2, 1 0.5 and 0.25 mg ml^−1^ by diluting 20 mg ml^−1^. Then, the solution of each concentration was sonicated and vortexed for 20 min, several times, to obtain a homogeneous colloidal suspension.

### 2.2. (C) Characterization

The structural characterization of iron oxide (*α*-Fe_2_O_3_) nanoparticles was investigated using a Philips X-ray diffractometer (XRD), Model: PW 3040-XPert PRO PANalytical. The XRD scan was performed on a powder sample for a 2*θ* angle range of 15–70 degrees at 40 kV and 30 mAusing CuK*α* radiation, (wavelength 1.54059 Å) with a scan step size of 0.0167o. Transmission Electron Microscopy (TEM), Model: TALOS F200 G2, FEI Company, USA, was used to examine samples' sizes and shapes. The TEM's working voltage was 200 kV. For TEM investigation the samples were dispersed in ethanol for 15 min before being drop-cast on an electron-transparent carbon-coated Cu grid and dried. We employed the Physical Property Measurement (PPMS) System, model: Inc.10307, Quantum Design, USA, which measured magnetization with the magnetic field ±9 T at the temperatures of 4 and 300 K. For ZFC measurements, the samples were cooled first at zero field and measurements were performed with the magnetic field during heating. For FC measurements, the samples were cooled from 400 K to 4 K with magnetic field, and magnetization measured in the presence of the same magnetic field during heating. There were five sets of FC/ZFC measurements by applying the magnetic fields of 50, 1000, 10 000, 30 000, 60 000 and 90 000 Oe. For each FC and ZFC measurements, we used the same magnetic field each time. FTIR spectroscopy, model: STA, 449 F3, Jupiter, UK, was used to get the Fourier Transform Infrared Spectroscopy observations. The FTIR spectra of uncoated and hematite-chitosan nanohybrid were collected in the 400–4000 cm*^−^*^1^ range. The hydrodynamic size and zeta potential of the samples were determined using dynamic light scattering (DLS) equipment, model ZEN 3600, Zetasizer, Malvern, UK. The measurements were performed at 25°C (room temperature), 37°C (human body temperature), and 45°C (hyperthermia temperature). The hydrodynamic size of hematite–chitosan nanohybrid at concentrations of 1.0 mg ml^−1^ was measured. Mössbauer spectroscopy, model: W302, USA, was used to determine the further magnetic characteristics. Mössbauer spectra were acquired using a transmission geometry resonant gamma-ray spectrometer in constant acceleration mode with a transducer velocity of 11 mm s^−1^. Before beginning the experiment, we calibrated the system using a metallic iron foil (99.999% purity) sample, and zero velocity was the centroid of the Mössbauer spectrum. We collected the Mössbauer spectra over 72 h at ambient temperature and zero magnetic field. A hyperthermia measurement system, the model: EASY HEAT 5060LI, Ambrell, USA, was used to analyze the heating profiles of the nanoparticle. The hyperthermia system consists of an 8-turns sample coil with a diameter of 4 cm. During the hyperthermia experiment, the coil current was 283 A, and the frequency of the coil signal was 343 kHz, generating a magnetic field of 26 mT in the sample coil. We measured temperature with time with 600 µl of sample in an Eppendorf tube of various concentrations of 0.25. 0.5, 1, 2, and 4 mg ml^−1^ with the AC magnetic field amplitude of 26 mT. The temperature of the sample was measured using a thermometer by stopping the magnetic field.

The specific loss power (SLP) can be explicitly related to the measured heating by

2.1 $$\mathrm{SLP}\;=\;\frac{C}{m}\frac{dT}{dt},$$

where *C* is the heat capacity of the solution sample (i.e. nanoparticles and suspending medium), m is the mass of the magnetic nanoparticle, and the temperature increment rate Δ*T*/Δ*t* was estimated from the initial slope in the linear range of temperature versus time curves. The heat capacity of the water, 4.18 J/g/°C, is considered the sample's heat capacity since the concentration of the magnetic nanoparticle is very small [41,42].

The cytotoxicity of the hematite–chitosan nanohybrid was investigated by introducing the sample solution (water as the solvent) into the Vero cell line, an African green monkey kidney epithelial cell line, and HeLa, a human cervical carcinoma cell line. Both were maintained in Dulbecco's Modified Eagles’ Medium (DMEM), which contained 1% penicillin–streptomycin (1 : 1) and 0.2% gentamycin, as well as 10% fetal bovine serum (FBS). 3 × 10^4^/200 µl cells were seeded onto 24 well plates and incubated at 37°C with 5% CO_2_. After 24 h, 50 µl of autoclaved samples were added to each well. Insoluble samples were washed out with fresh media after 48 h of incubation, and cytotoxicity was assessed using a hemocytometer and an inverted light microscope. For each sample, duplicate wells were used. A sonicator, Model: Power Sonic 510, Hwa Shin Technology, Seoul, Korea, was used to equally disperse nanoparticles in liquids. The input voltage was 230 volts at 50 hertz, while the output power was 500 watts.

## 3. Results and discussions

**Figure 1.**
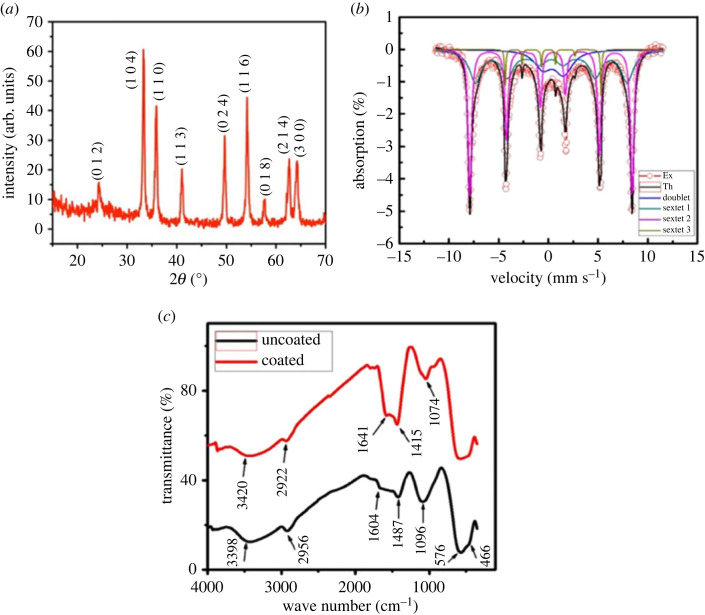
(*a*) X-ray diffraction pattern of iron oxide (*α*-Fe_2_O_3_) nanoparticle (uncoated), (*b*) Mössbauer spectrum of iron oxide (α-Fe_2_O_3_) nanoparticle, (*c*) FTIR spectra of uncoated (black) and hematite–chitosan nanohybrid (red).

Figure 1*a* shows the XRD patterns of hematite nanoparticles. The strong and sharp diffraction peaks indicate the high crystallinity of these samples [43]. The XRD data also revealed that the *α*-Fe_2_O_3_ obtained had a rhombohedral structure. The significant peaks appearing at the 2*θ* range of 24.16°, 33.12°, 35.63°, 40.64°, 49.47°, 54.08°, 57.42°, 62.71° and 64.29° can be ascribed to the (012), (104), (110), (113), (024), (116), (018), (214) and (300) crystalline structures corresponding to pure *α*- Fe_2_O_3_ nanoparticles (JCPDS-ICDD 89–0596). No other diffraction peaks corresponding to ferrite nitrite or other iron oxides, such as Fe_3_O_4_ and *γ*-Fe_2_O_3_, can be observed, but those of *α*-Fe_2_O_3_, thus suggesting the high phase purity of the as-synthesized products.

The grain size was calculated using Modified Debye Scherrer's formula [44].

3.1 $$\ln\beta =\ln\frac{K\lambda }{D}+\ln\left(\frac{1}{\cos\theta }\right),$$

where *D* = grain size, *K* = a dimensionless shape factor with a value of unity. Here, *K* = 0.94, = 1.54060 Å for Cu-K*α* radiation, = FWHM (full-width half maxima) of the peak of radiation in radian. By plotting ln against ln $\left(\frac{1}{\cos\theta }\right)$ the grain size can be found. The calculated grain size was 27 nm. The lattice constants (a and c) measured for the *α*-Fe_2_O_3_ nanoparticles were *a* = 5.07 Å and *c* = 13.68 Å which were calculated by using the following equation [45]:

3.2 $$\frac{1}{d^{2}}=\frac{h^{2}+k^{2}}{a^{2}}+\frac{l^{2}}{c^{2}}$$

where *d* is the interplanar distance (d-spacing) and (hkl) are the miller indices. The values of the lattice constants match well with the previously recorded values by Rasheed *et al*. [45] and with (JCPDS, Ref. Code: 33-0664).

Figure 1*b* shows the Mössbauer spectra of the uncoated iron oxide (*α*-Fe_2_O_3_) nanoparticles. It can be observed from the figure that there is a mixture of doublet and sextets. The doublet pattern occurs due to the interaction of gamma rays with the electric field of the electrons of the sample, which indicates the superparamagnetic behaviour of the nanoparticles. The sextet pattern arose from the interaction of gamma-ray with the magnetic field of the hematite. Using the models fitting of the experimental spectrum provides the hyperfine interaction parameters, such as isomer shift, quadrupole splitting and hyperfine magnetic field, and presented in table 1.

**Table 1.** Hyperfine interaction parameters of Mössbauer spectroscopy acquired at room temperature and zero-field conditions.

obs. peak no	pattern	isomer shift mm s^−1^	quadrupole splitting mm s^−1^	hyperfine field kOe	area
1	doublet	0.216	2.103	17.747	0.166
2	sextet 1	0.300	0.000	474.070	0.426
3	sextet 2	0.365	1.710	499.392	0.491
4	sextet 3	0.253	1.810	284.028	0.044

From figure 1*b* and table 1 we notice the existence of slow relaxation to be more prominent because of the longer relaxation than the time scale of Mössbauer spectroscopy. S. Mørup *et al*. [46] conducted Mössbauer studies on interacting and noninteracting *α*-Fe_2_O_3_ nanoparticles and provided a detailed account of the magnetization process. The magnetic energy on particle *i* with the neighbouring atoms *j* is

3.3 $$E_{i}\;=\;KV_{i}sin^{2}\theta -M_{i}\sum_{j}K_{ij}M_{ij}.$$

Here, *K_ij_* is the exchange coupling constant between the particles *i* and *j*, and *M_i_* and *M_j_* are the sublattice magnetization of the particles *i* and *j*. The first part of the equation represents the contribution to noninteracting particles and the second part represents the contribution arising from particles with wide interaction. Since the Mössbauer spectrum presented in figure 1*b* is for the sample without coating, it is expected that strong interparticle interactions exist and therefore, the second part of equation (3.3) becomes predominant. While at low temperature there is only one energy minima, at finite temperature, the sublattice magnetization fluctuate around the energy minima. In the uncoated samples, the iron oxide nanoparticles are randomly packed and therefore there exists a broad distribution of exchange coupling constants, which gives rise to the different order parameters in the different parts of the sample [46]. These give rise to the assymmetric line broadening in the sextet and a mixed slow fast relaxation in figure 1*b*. It is also shown [46] that magnetic anisotropy energy drops by a factor of 10 in the particle size range of 6–25 nm. In this study, average particle size was obtained approximately 27 nm with a distribution in particle size. Therefore, we can expect mixed relaxation as obtained in figure 1*b* and table 1. Bødker *et al*. [12] also obtained the existence of size distribution of hematite nanoparticles leading to the distribution of anisotropy energy, which contributes to magnetic energy and to the sextet contribution.

Chitosan–hematite nanohybrids have electrostatic interaction due to the presence of the –OH group on the surface, and chitosan is positive in nature (protonated). Figure 1*c* shows Fourier transform infrared spectra (FTIR) of uncoated and chitosan-coated *α*-Fe_2_O_3_ nanoparticles in the range of 400–4000 cm*^−^*^1^, which confirmed the bonding of chitosan with hematite nanoparticles by the peak shifts in the two spectra. We assigned the broad band at 3398 cm*^−^*^1^ to the O–H stretching vibration. We also assigned the absorption bands around 1604 cm*^−^*^1^ and 1487 cm*^−^*^1^ due to the asymmetric and symmetric bending vibration of C=O and an absorption band at 1096 cm*^−^*^1^. The bands at 576 and 466 cm*^−^*^1^ correspond to the Fe–O stretching and bending modes of *α*-Fe_2_O_3_, respectively. The peak positions reported in figure 1*c* coincide precisely with those reported by Farahmandjou *et al*. [47]. Further, M. Tadic *et al*. [48] synthesized *α*-Fe_2_O_3_ nanoparticles by hydrothermal method to study the magnetic and structural properties of the hematite nanoparticles. They also observed two absorption peaks at about 515 and 430 cm*^−^*^1^ corresponding to stretching and bending modes of the Fe–O bond in hematite.

**Figure 2.**
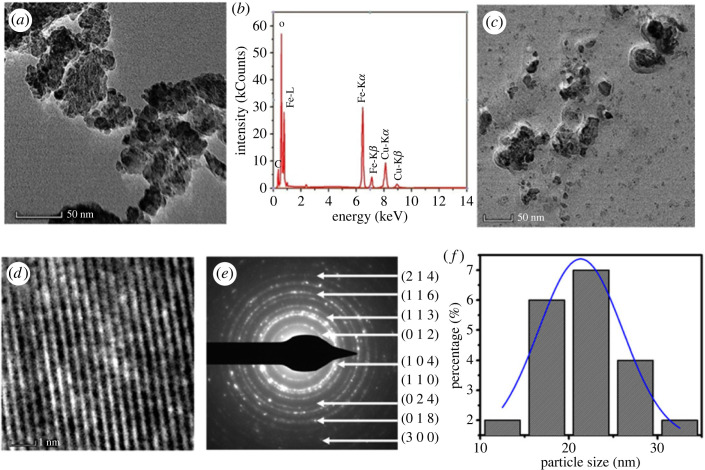
TEM micrographs of the (*a*) uncoated α-Fe_2_O_3_ nanoparticles, (*b*) EDS spectrum of uncoated *α*-Fe_2_O_3_ nanoparticle (*c*) hematite–chitosan nanohybrid, (*d*) the HRTEM image which demonstrates the lattice fringe of crystalline particles, (*e*) SAED patterns *α*-Fe_2_O_3_ nanoparticles, (*f*) size distribution of *α*-Fe_2_O_3_ nanoparticles.

Hematite–chitosan nanohybrid demonstrates the bonding of chitosan with *α*-Fe_2_O_3_ by the peak shift of Fe–O at 576 and 466 cm*^−^*^1^ in the spectrum of uncoated samples. The peak at 1641 cm*^−^*^1^ represents the N–H vibration, 1074 cm*^−^*^1^ represents the C–O bond, and the peak at 1415 cm*^−^*^1^ represents the C–N vibration of the amino group. The FTIR spectra of chitosan-coated iron oxide (*α*-Fe_2_O_3_). They also reported EM bright-field images were acquired to analyze the size and morphology of the uncoated nanoparticles, their corresponding EDS spectrum, and the dispersion of the coated nanoparticles, which are presented in figure 2*a–c*, respectively. The average size of the nanoparticles in TEM was about 25 nm, which is almost similar to the average size obtained by using XRD. Despite the significant agglomeration, most particles appeared spherical in the TEM image, and some particles were slightly non-spherical. The particle size distribution is shown in figure 2*f*. Tadic *et al.* [49] also prepared hematite nanoparticles using the hydrothermal route and found the average size to be approximately 8 nm, which is considerably smaller than the present study. They also reported uniform nanoparticles of spherical morphology with narrow size distribution. To study the elemental composition of *α*-Fe_2_O_3*,*_ Electron Dispersive X-ray Spectroscopy (EDS) analysis was performed which shows the peaks Fe and O of hematite. The relative amounts of Fe and O were found to be 25 and 56 in atom %, respectively.

A high-resolution transmission electron microscopy (HRTEM) image of the sample is presented in figure 2*d*, demonstrating that the spherical nanoparticles are highly crystallized as the lattice fringes can be clearly seen in the figure. The HRTEM image was acquired at the magnification of 1.06 M. The image presented in figure 2*d* was further zoomed in (the scale-bar on the figure refers to understand the actual size). Selected area electron diffraction patterns (SAED) of the uncoated and chitosan-coated nanoparticles are presented in figure 2*e*. Corresponding d-values of all the diffraction rings in the electron diffraction patterns belong to the *α*-Fe_2_O_3_ phase, which are indexed in figure [49]. The size distribution was determined by Image J software and presented in figure 2*f*. The size distribution follows the lognormal distribution. Several authors synthesized *α*-Fe_2_O_3_ or hematite nanoparticles by the hydrothermal method and reported in the literature. Tadic *et al*. [48] observed irregular morphology with sizes around 50 nm without any surfactant. Khalil *et al*. [50] obtained the particle size of around 100 nm using TEM. Pantharee *et al*. [51] found spherical particles with the size in the range 15–205 nm depending on the surfactant type and concentration. Li *et al*. [52] synthesized *α*-Fe_2_O_3_ nanocrystals of spectacular morphologies of hollow nanoolives, nanotubes, nanospindles and nanoplates. In this study, we obtained semispherical morphology with an average particle size of around 25 nm.

**Figure 3.**
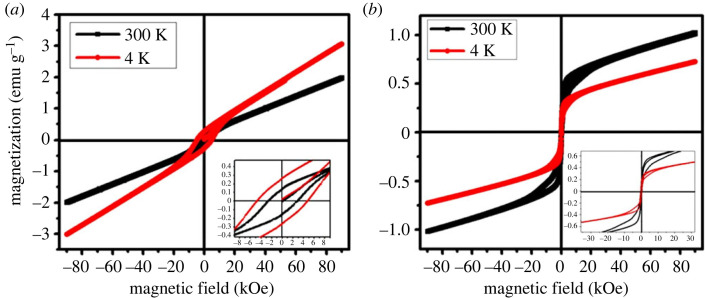
(*a*) *M*–*H* curves of the uncoated and (*b*) hematite–chitosan nanohybrid at 300 and 4 K with a maximum applied field of 90 kOe.

Figure 3*a* shows the M(H) curves of the *α*-Fe_2_O_3_ nanoparticles in the uncoated stage at 4 and 300 K with the magnetic field of ±9T. Existence of the hysteresis area suggests the ferrimagnetic contribution. The loop area is larger at 4 K and the coercivity and remanent magnetization values are H*_C,_*_4*K*_ = 4762 Oersted, *M_r,_*_4*K*_ = 0.269 emu g^−1^, respectively, while the maximum magnetization at 9T is 3.00 emu g^−1^. The M(H) curve at 300 K suggests a reduction in the hysteresis loop area, demonstrating an increase in the superparamagnetic contribution. The mixed ferrimagnetic and superparamagnetic contributions at 300 K were also confirmed by Mössbauer spectroscopy in figure 1*b*. The maximum magnetization at 300 K, *M*_max,300 K_ = 1.98 emu g^−1^, the coercivity *H*_C*,*300*K*_ = 2777 Oersted and remanent magnetization *M*_r*,*300*K*_ = 0.150 emu g^−1^, which are smaller compared to the values at 4 K. Usually, the magnetic properties of a material depend on several factors, including crystal structure, particle size, and morphology, as well as the competition between magnetic and thermal energy. Previous research showed that hematite nanoparticles are likely to exhibit a wide range of magnetic characteristics between superparamagnetism, antiferromagnetism, and ferromagnetism, with the slight fluctuation in particle sizes [14,46,53,54]. Bødker *et al.* [14] and Mørup *et al*. [46] reported an elaborate illustration of the effect of particle size on the magnetic properties of hematite nanoparticles (particle size around 16–17 nm) by the Mössbauer spectroscopy and magnetization studies. The nature of the hysteresis loops reported by them agrees with our studies. They demonstrated from the *M*–*H* measurements that the magnetization consists of both ferromagnetic and superparamagnetic contribution at room temperature. Sharma *et al*. [55] also observed superparamagnetic properties of hematite nanoparticles and an increase of magnetization in smaller nanoparticles while studying size-dependent magnetic properties. Xu *et al*. [56] found the superparamagnetic state while preparing hematite nanoplates with a length approximately 100 nm, a width approximately 30 nm, and a thickness below 10 nm. Figure 2*f* shows the size distribution of nanoparticles in this study from TEM image. This wide distribution of particle size may contribute to the mixed ferrimagnetic and superparamagnetic contributions in the magnetic properties [53–57].

Our studies match well with the observations reported previously by Tadic *et al*. [49] . They found the existence of ferromagnetism in *α*-Fe_2_O_3_ nanoparticles at 10 K temperature, and the nanoparticles showed a transition from ferromagnetism to superparamagnetism at 300 K (room temperature). They obtained bimodal distribution of particle size, and elucidated that the particle size smaller than 10 nm produced blocking temperature and superparamagnetism and particles around 20 nm produced Morin transition. In the present study, we reported in figure 2*f* a distribution in particle size. Therefore, our assumption is at 4 K, magnetization consists of the contributions of antiferromagnetic and weak ferromagnetic. At room temperature, the contributions in magnetization emanate from superparamagnetic and weak ferromagnetic. The prepared hematite nanoparticles have a high magnetization of 1.98 emu g^−1^ at 300 K, which is significantly greater than the magnetization of bulk *α*-Fe_2_O_3_ materials (*M*_max_ = 0.3 emu g^−1^) [10]. This high magnetization is desirable for magnetic hyperthermia applications—a promising cancer treatment therapy [32,33].

The magnetic anisotropy can be calculated from the following formula [6,58]:

3.4 $$H_{c}=\frac{2K_{1}}{M_{s}},$$

where *K*_1_ = magnetic anisotropy, *M*_S_ = saturation magnetization and *H*_C_ = coercivity. The value of the magnetic anisotropy of the uncoated samples, *K*_1_ for the *α*-Fe_2_O_3_ nanoparticles at 300 K is 0.51 × 10^5^ erg cm^−3^, and at 4 K the magnetic anisotropy, *K*_1_ = 1.35 × 10^5^ erg cm^−3^. This large anisotropy causes interparticle interactions of both exchange and dipolar interactions. When the particles are coated with nonmagnetic chitosan the long-range dipolar interactions are eliminated [14]. Roberts *et al*. [59] demonstrated that the single domain threshold size of hematite is about 25–30 nm. According to this study, we can predict that most of the particles after coating are of a single domain, although some multi-domain particles of larger size exist.

Figure 3*b* shows the field dependence of magnetization (*M*–*H*) curves of the hematite–chitosan nanohybrid at *T* = 4 and 300 K with the magnetic field sweeping from −9T to +9T.

The difference in the shape of the M–H curves in figure 3*a* and *b* is striking. This is because coating reduces interparticle long-range interactions. A large anisotropy determined in the previous section would reduce to a great extent when the particles mostly become single domain by coating with nonmagnetic chitosan, which acts as the barrier for interparticle interactions. Therefore, the ease of magnetization for the coated samples is greater than for uncoated sample because of the reduction of anisotropy. As a result, coercivity and remanence are reduced significantly for coated samples compared to the uncoated samples. Further, when chitosan-coating acts as a barrier for interparticle interactions superparamagnetic/ferromagnetic blocking becomes more prominent than the Morin transition [14,46]. The distribution of particle size gives rise to the distribution of blocking temperature. The M–H curves both at 4 K and 300 K presented in figure 3*b* indicate weak ferromagnetic behaviour. The magnetization has not saturated at the maximum field of 90 kOe because of the canted spin. The saturation magnetization of the sample was *M*_max*,* 300*K*_ = 1.02 emu g^−1^ and *M*_max, 5*K*_ = 0.73 emu g^−1^. The lower value of *M*max at 5 K than 300 K is because the spins are in the blocked stage at 5 K. Again, these coated nanoparticles have a higher magnetization than the previously reported magnetization of bulk *α*-Fe_2_O_3_ materials (*M*_max_ = 0.3 emu g^−1^) [10]. The values of M*_s_* are smaller for the coated samples than the uncoated samples because of the absence of long-range dipolar interactions due to coating [14] as explained above.

**Figure 4.**
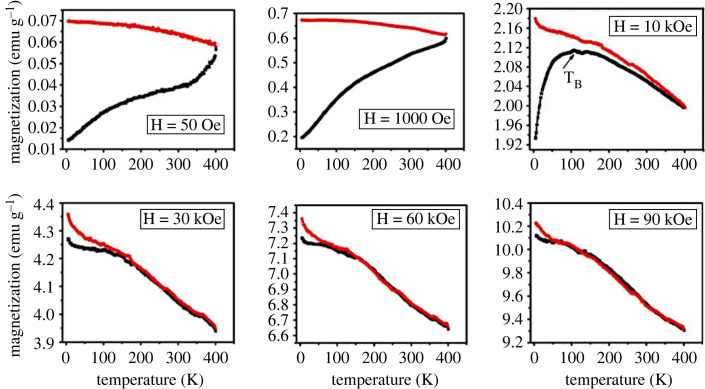
Field-cooled (FC) and zero-field cooled (ZFC) of uncoated *α*-Fe_2_O_3_ nanoparticles with the applied field of 50 Oe, 1000 Oe, 10 kOe, 30 kOe, 60 kOe and 90 kOe.

Figure 4 shows field-cooled (FC) and zero-field-cooled (ZFC) curves for *α*-Fe_2_O_3_ with the applied magnetic fields of 50, 1000, 10 000, 30 000, 60 000, 90 000 Oe in the as-dried condition. It is evident from figure 4 that the ZFC magnetization curve with 50 Oe applied field bears several kink points, which might be related to the blocking temperature and Morin transition. Tadic *et al*. [60] found the Morin transition at 225 K for particle size ∼40 nm [51]. They also reported that Morin transition shifts to higher temperatures for increasing particle size. However, the Morin transition remains undetected for particles having sizes below approximately 10 nm [60]. From TEM studies in figure 2*f*, we found a broad distribution of particle size. Therefore, the transition temperature manifested in the ZFC curve in figure 4 with a 50 Oe applied field shows a wide range. We assume that the kink point around 100 K represents the blocking temperature and the kink point around 250∼260 K represents the Morin transition [60]. In a separate work, M. Tadic *et al*. [57] also found similar observations for particles having sizes around 20 nm with applied field H = 100 Oe. Although there is no direct relation of Morin transition to the hyperthermia study, transition temperatures are crucial to understanding the magnetic state of the nanoparticle at room temperature. We discussed that below the Morin transition, the *α*-Fe_2_O_3_ nanoparticles are antiferromagnetic, and above the Morin transition temperature, the *α*-Fe_2_O_3_ nanoparticles are ferromagnetic. The Morin transition at around 250–260 K in the present study confirms the magnetic state of the nanoparticles as weak ferromagnetic at room temperature, which has significance in the hyperthermia studies reported later. The kink-point representing Morin transition is also appreciable in the FC and ZFC curve measured with 1000 Oe, while at the higher fields Morin transition is obscure. It is also evident from figure 4 that, for H = 50 Oe, the branches of ZFC and FC magnetization do not join up to T_irr_ = 400 K (irreversibility temperature), which is due to the effect of magnetic and shape anisotropy as evident in figure 3 with 50 and 100 Oe. Since the applied field (*H* = 50 and 100 Oe) is much lower than the anisotropy field (approx. 2777 Oe), the anisotropy energy is dominant here, and the blocking temperature is not sharp for these lower applied fields. The FC magnetization increases gradually on cooling from 400 to 4 K, whereas the ZFC curve shows a considerable divergence. Tadic *et al*. [60] observed similar patterns with T_irr_ > 400 K.

When the applied field is *H* = 10 kOe in figure 4, a blocking temperature at about *T*_B_ = 106 K was noticed that originated from the *α*-Fe_2_O_3_ nanoparticles in the ZFC curve. Here, the applied field is close to the anisotropy field. Thus, initially, the magnetic moment increased with decreasing temperature since with the decrease of thermal energy, exchange energy dominates, which increases magnetization up to the blocking temperature (*T*_B_) of 355 K. The ZFC curve shows magnetization decreases sharply below the blocking temperature (*T*_B_) as the anisotropy energy dominates below *T*_B_. On the contrary, the FC magnetization increases continuously even below T*_B_* reaching down to 4 K. Tadic *et al*. confirmed that this behaviour of the FC magnetization is due to exchange energy overcoming the anisotropy energy in the FC condition [61,62]. Near the blocking temperature, the sample showed a ferromagnetic nature. The temperature at which the ZFC and FC curves begin to separate was mentioned as irreversibility temperature in some literature [63–65].

For *H* = 30 kOe, the applied field is much higher than the anisotropy field. So, the magnetization increases with decreasing temperature for both FC and ZFC conditions because the exchange energy overcomes the anisotropy energy. Similar results are observed for the ZFC-FC data of the sample acquired at 60 kOe, and 90 kOe. For *H* = 30 kOe, *T*_irr_∼172 K, for *H* = 60 kOe, *T*_irr_∼55 K, and for *H* = 90 kOe, *T*_irr_∼38 K. It can be observed that the irreversibility between ZFC and FC magnetization depends greatly on the applied magnetic field. Thus, as the applied magnetic fields are increased, the ZFC and FC curves converge at a relatively lower temperature because at higher magnetic fields exchange energy is stronger which overcomes the anisotropy energy barrier of larger particles. Further, it is interesting to note that even with 90 kOe, the convergence of FC and ZFC was not possible because of the existence of small volume of particles with high anisotropy energy that was not possible to overcome by the exchange energy. It was pointed out by S. Mørup *et al*. [46] that as the particle size decreases from 25 to 6 nm the anisotropy increases by a factor of 10.

**Figure 5.**
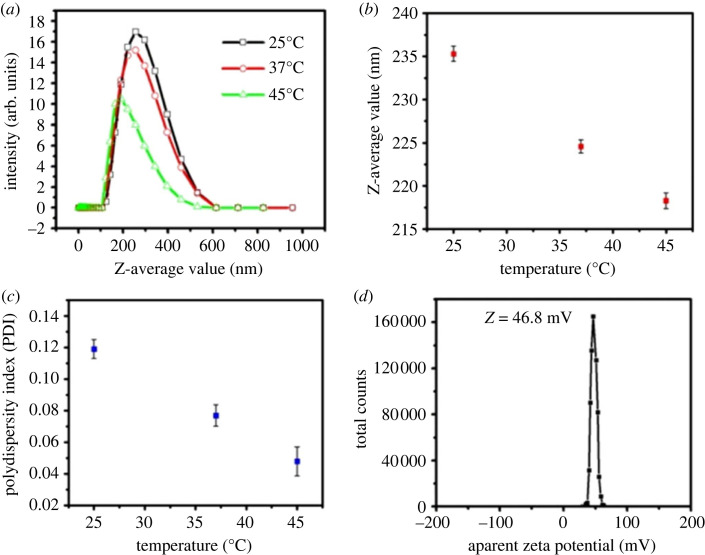
(*a*) Intensity versus z-average traces acquired from dynamic light scattering of hematite–chitosan nanohybrid at the temperatures of 25, 37 and 45°C, (*b*) the variation z-average with temperature, (*c*) polydispersity index (PDI) with temperature, (*d*) zeta potential distribution for chitosan-coated iron oxide (*α*-Fe_2_O_3_) nanoparticles.

Dynamic Light Scattering (DLS) is a technique for measuring the size of particles and molecules in suspension. In this work, the average hydrodynamic diameter and polydispersity index (PDI) values of the hematite-chitosan nanohybrid of concentrations 0.25, 0.5, 1.0, 2.0 and 4.0 mg ml^−1^ were measured at 25°C (room temperature), 37°C (body temperature) and 45°C (hyperthermia temperature) which are presented in figure 5*a*. The values of the hydrodynamic diameter and polydispersity index acquired from this figure are presented in table 2. The hydrodynamic diameter of the coated magnetic nanoparticle is crucial for its application as a hyperthermia agent. Because of the zeta potential, surface coating and ionic strength, the nanoparticle's hydrodynamic size can be 5–20 times or even higher when dispersed in water [66,67]. P Roychoudhury *et al*. observed the range of hydrodynamic diameter for biomedical applications should be less than 250 nm [68].

**Table 2.** Average hydrodynamic diameter and polydispersity index (PDI) of the hematite–chitosan nanohybrid of concentrations 0.25, 0.5, 1.0, 2.0 and 4.0 mg ml^−1^ at 25°C (room temperature), 37°C (body temperature) and 45°C (hyperthermia temperature).

temperature	hydrodynamic diameter	(PDI)
25	235.3	0.119
37	224.6	0.077
45	218.3	0.048

The polydispersity index (PDI) describes particle variation, which may occur due to the agglomeration of the sample during analysis. PDI value in the range 0.1 to 0.4 indicates a moderate polydisperse distribution [69]. For biomedical applications, the PDI value must be less than 0.35 [70]. Figure 5*b* and *c* illustrate the variation of hydrodynamic diameter and PDI with temperature, respectively. Both the hydrodynamic diameter and PDI value decrease with increasing temperature. The values of hydrodynamic diameter were between 218 and 235 nm, and the values of PDI were between 0.048 and 0.119. This confirms that the hydrodynamic size and PDI value remained within the limit for biomedical applications for every temperature. Gozde Unsoy *et al*. also observed the hydrodynamic diameter between 58 and 103 nm for chitosan-coated iron-oxide nanoparticles [71].

Zeta potential is considered one of the essential tools for perceiving the state of the nanoparticle surface and anticipating the long-term stability of the magnetic nanoparticle. Figure 5*d* presents the zeta potential distribution of chitosan-coated iron oxide (*α*-Fe_2_O_3_) nanoparticles of 1.0 mg ml^−1^ concentration. The zeta potential was obtained as +46.8 mV. As per the literature, particles with zeta potential larger than ±35 mV have excellent stability, whereas between −10 mV and +10 mV, will experience rapid agglomeration unless they are sterically protected [72]. The zeta potential of the chitosan-coated iron-oxide nanoparticles observed by Shi *et al*. [73] was +47.8 mV, which is close to the zeta potential of present study.

**Figure 6.**
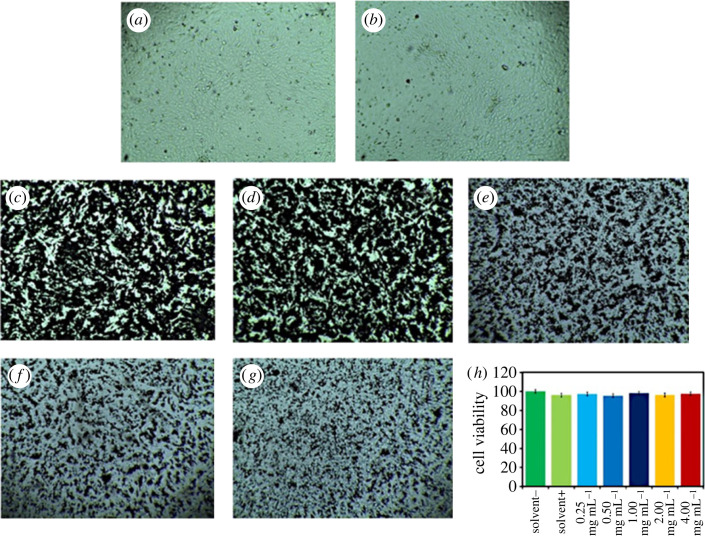
Cytotoxicity studies of hematite–chitosan nanohybrid on Vero cells (*a*) sample with solvent, (*b*) sample without solvent (*c*) 4 mg ml^−1^ concentrated sample, (*d*) 2 mg ml^−1^ concentrated sample, (*e*) 1 mg ml^−1^ concentrated sample, (*f*) 0.5 mg ml^−1^ concentrated sample, (*g*) 0.25 mg ml^−1^ concentrated sample, (*h*) %viability of Vero cells at different concentrations.

Cytotoxicity of the hematite-chitosan nanohybrid was examined by applying the sample solution (water as solvent) into the Vero cell line, which is a kidney epithelial cells extracted from an African green monkey. We used Vero cell lines to study the viability assay on healthy cell lines. The cell images used as control with/without solvent are shown in figure 6*a* and *b*, respectively. The cell images incubated with the coated samples for 24 h with the concentrations of 4, 2, 1, 0.5 and 0.25 mg ml^−1^ are presented in figure 6*c–g*, respectively. Figure 6*h* depicts % viability of Vero cells at different concentrations. The bar chart shows that the survival of the cell was more than 90%. This confirms that the hematite–chitosan nanohybrid were viable for the Vero cell lines.

**Figure 7.**
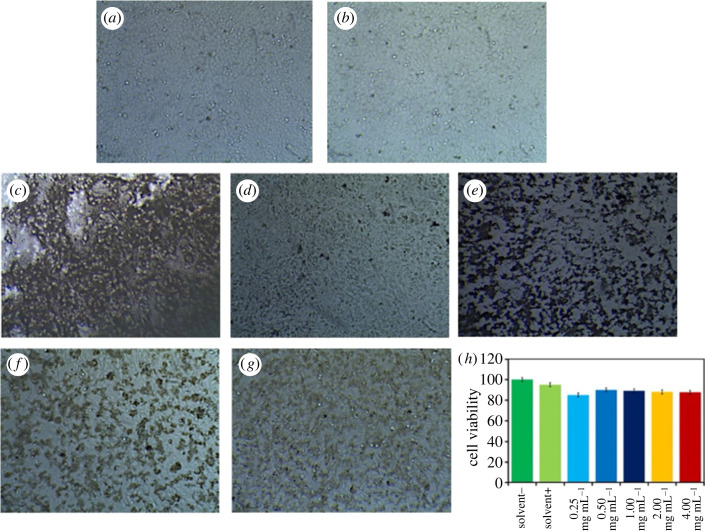
Cytotoxicity studies of hematite–chitosan nanohybrid on HeLa cells (*a*) sample with solvent, (*b*) sample without solvent (*c*) 4 mg ml^−1^ concentrated sample, (*d*) 2 mg ml^−1^ concentrated sample, (*e*) 1 mg ml^−1^ concentrated sample, (*f*) 0.5 mg ml^−1^ concentrated sample, (*g*) 0.25 mg ml^−1^ concentrated sample, (*h*) %viability of HeLa cells at different concentrations.

To examine the cytotoxicity of the cancer cells, the hematite–chitosan nanohybrid was examined by applying chitosan-coated hematite nanoparticles to the HeLa cell line. The cell images of the control, i.e. HeLa cell lines with/without the solvent are shown in figure 7*a* and *b*, respectively. Figure 7*c–g* demonstrates the images of cells incubated with the coated sample for 24 h. The concentrations used in this study were also 4, 2, 1, 0.5 and 0.25 mg ml^−1^. Figure 7*h* presents the % survival of the cells with the concentrations of chitosan-coated nanoparticles, which shows viability. The results show that the chitosan-coated hematite does not have any chemical toxicity on the cancer cells, which has the promise that the cancer cells will annihilate only by rf induction heating in the presence of chitosan–hematite nanohybrid.

**Figure 8.**
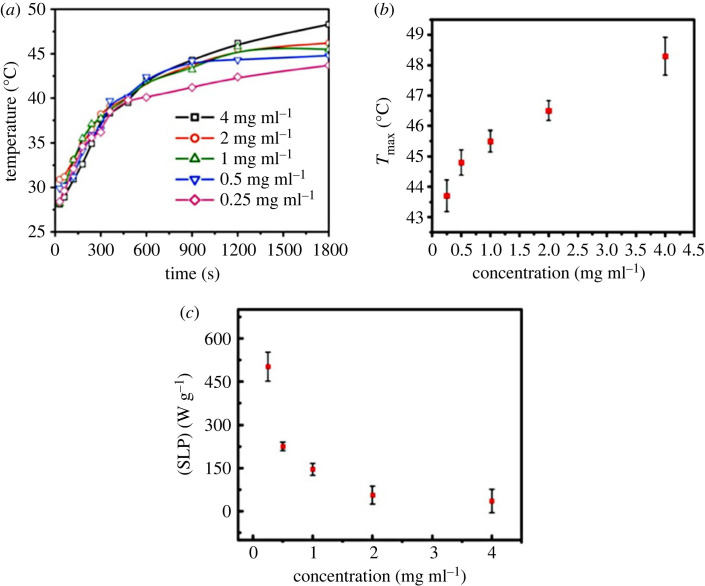
(*a*) Time dependence temperature of hematite–chitosan nanohybrid at different concentrations while exposed to rf magnetic field (lines are only to guide eyes), (*b*) maximum temperature acquired from (*a*) and (*c*) specific loss power determined from the initial slope of (*a*) with the variation of concentration.

Figure 8*a* shows rf induction heating properties as a function of time for chitosan-coated (*α*-Fe_2_O_3_) nanoparticles. To study the sample's heating capacity, different concentrations of chitosan-coated samples were experimented. The increase in temperature showed variation over a wide range of temperatures, and several authors have reported that the concentrations of the particles can play a key role [20,74,75]. Hence, the heating properties of the chitosan-coated (*α*-Fe_2_O_3_) nanoparticles were studied by varying their concentrations.

The heat generated at all concentrations (0.25, 0.50, 1, 2 and 4 mg ml^−1^) is presented graphically in figure 8*a*. The maximum temperatures obtained by the solutions of concentrations 4, 2, 1, 0.5 and 0.25 mg ml^−1^ are 48.3°C, 46.5°C, 45.5°C, 48.8°C and 42.9°C, respectively. The temperature should be in the range of 42–46°C to kill cancer cells by hyperthermia [76,77]. The temperature attained by the chitosan-hematite nanohybrid solution of the lowest concentration of 0.25 mg ml^−1^ is 42.9°C, i.e. within the hyperthermia range. The temperature attained by the concentrations of 0.5 mg ml^−1^ or above is well-within the hyperthermia range. Similar behaviour was observed by S. M. Hoque *et al*. [74] with the heating efficiency of chitosan- and PEG-coated NiFe_2_O_4_ particles. They obtained the maximum temperature attained by 1.00 mg ml^−1^ concentration was about 37.7°C, which can be compared with the results found in this study. We observe that the maximum temperature attained by *α*-Fe_2_O_3_ nanoparticle at 1 mg ml^−1^ is 45.5°C. It is counterintuitive since NiFe_2_O_4_ is ferrimagnetic and *α*-Fe_2_O_3_ is paramagnetic/weak ferromagnetic. Again, this is fascinating because iron-based compounds are always more plausible for biomedical applications.

Magnetic hyperthermia is based on applying Alternating Magnetic Field (AMF). When in a magnetic material, AMF is applied, the magnetization of the magnetic materials is aligned in one direction. When the AMF is reversed, the change of magnetic moment against internal resistance forces releases heat into the environment (the so-called Hysteresis loss, Brownian and Neel relaxation process). Research has shown that this heat can damage and kill cancer cells [74]. Since superparamagnetic particles produce higher hysteresis losses for monodomain magnetic particles, they generate more heat than ferrimagnetic ones under the same conditions. Figure 8*b* presents the maximum temperature *T*_max_ attained by the hematite–chitosan nanohybrid at different concentrations that can be used as the calibration curve. The maximum temperature (*T*_max_) increases with the increase of concentration. From the calibration curve, the required temperature can be attained by tuning the concentration to avoid possible overheating.

The specific loss power (SLP) is defined as electromagnetic power absorbed per mass unit of magnetic materials and is expressed in watts per kilogram. When magnetic nanoparticles (MNPs) are exposed to external alternating magnetic fields, the variation of the specific loss power with the anisotropy constant shows exponential rise, and the distribution of the specific loss power with the anisotropy constant shifts with temperature. Furthermore, the specific loss power determines how quickly the temperature rises in MNPs used for hyperthermia.

As a result, the specific loss power is the engineering parameter that governs the efficacy of hyperthermia [78]. The specific loss power in this study evaluated through equation (1.1) are presented in figure 8*c*. The SLP of the solutions of concentrations 4.0 mg ml^−1^, 2.0 mg ml^−1^, 1.0 mg ml^−1^, 0.5 mg ml^−1^, 0.25 mg ml^−1^ are 35.53 W g^−1^, 56.43 W g^−1^, 146.3 W g^−1^, 225.72 W g^−1^, 501.6 W g^−1^, respectively. Figure 8*c* reveals that the SLP of concentration 0.25 mg ml^−1^ is the highest with a value of 501.6 W g^−1^, while the SLP of concentration 4.0 mg ml^−1^ is the lowest, 35.53 W g^−1^; thus, the SLP decreases with the increase of concentrations. Decreasing the value of SLP with the increase in concentration was also observed in the previous studies [78–81]. The specific loss power and the maximum temperature, *T*_max_, in figure 8 are remarkable, when we consider the magnetization as *M*_max, 300 K_ = 1.98 emu g^−1^ with the applied field 9 T in this study. We believe that the higher anisotropy of *α*-Fe_2_O_3_ played a significant role in contributing higher values of SLP and *T*_max_. This is because Néel relaxation has a exponential dependence on the anisotropy constant by the relation $\tau_{N}=\tau_{o}e^{KV/k_{B}T}$. Although anisotropy doesn't have any effect on Brownian relaxation, but anisotropy increases hysteresis loss. Combined increase of Néel relaxation and hysteresis loss with the limited increase of magnetic anisotropy can contribute to increasing the specific loss power and *T*_max_. If magnetic anisotropy is too high, Néel relaxation cannot occur. In the previous study by Hoque *et al*. [82], ZnFe_2_O_4_ possessing the magnetization of 13.4 emu g^−1^ with the applied field of 2 T yielded *T*_max_ of around 37°C with a very high concentration of 16 mg ml^−1^. S. Yoon *et al*. [83] determined the temperature dependence of the magnetic anisotropy constant of ZnFe_2_O_4_ with the particle size of 8.5 nm. They obtained the value of magnetic anisotropy constant 11 000 J m^−3^ at 4.2 K, which dropped to a value of 200 J m^−3^ at 60 K. The magnetic anisotropy constant of ZnFe_2_O_4_ is negligible at room temperature as evidenced from the findings of Yoon *et al*. [83]. Mørup *et al*. [46] determined particle size dependence of magnetic anisotropy constant from Mössbauer spectroscopy and found that the anisotropy constant decreases by a factor of 10 in the particle size range of 6–25 nm. In our study we reported above that the anisotropy constant is 5000 J m^−3^ for the uncoated samples at room temperature, which is close to the value of S. Mørup *et al*. [46] in the particle size range of 27 nm obtained in this study. The higher values of SLP in the range of 35–501 W g^−1^ for 0.25–5 mg ml^−1^ concentration range and *T*_max_ in the range of 42.9–48.3°C are due to the higher anisotropy of α-Fe_2_O_3_ nanoparticles in this study although very small magnetization of 1.98 emu g^−1^.

## 4. Conclusion

In this research, we developed chitosan–hematite nanohybrid synthesized by the hydrothermal method that yielded well-crystalline hematite nanoparticles of 27 nm size. The functionalization of hematite with chitosan was successful, proved by FTIR, TEM and PPMS studies. The nanohybrids are viable for both healthy and cancer cells, which is further assurance for the applications. The maximum temperature, *T*_max_, in the range of 42.9–48.3°C and specific loss power 35–500 W g^−1^ are remarkable for the concentration range of 0.25–4 mg ml^−1^ when the maximum magnetization was 1.98 emu g^−1^ with 90 kOe applied field. The *T*_max_ and SLP at concentrations as low as 0.25–0.5 mg ml^−1^ of chitosan–hematite nanohybrids open the doors of new possibilities in hyperthermia studies.

## Data Availability

This article has no additional data.
